# Aspartame and Its Microhydrated Aggregates Revealed by Laser Spectroscopy: Water–Sweetener Interactions in the Gas Phase

**DOI:** 10.1021/acs.jpca.4c04315

**Published:** 2024-08-02

**Authors:** Paul Pinillos, Ander Camiruaga, Fernando Torres-Hernández, Pierre Çarçabal, Imanol Usabiaga, José A. Fernández, Rodrigo Martínez

**Affiliations:** † Department of Physical Chemistry, Faculty of Science and Technology, University of the Basque Country (UPV/EHU), B° Sarriena S/N, Leioa 48940, Spain; ‡ Institut des Sciences Moléculaires d’Orsay (ISMO), Université Paris Saclay, CNRS, Orsay 91405, France; § Department of Chemistry, Faculty of Science and Technology, University of La Rioja, Madre de Dios 53, Logroño 26006, Spain

## Abstract

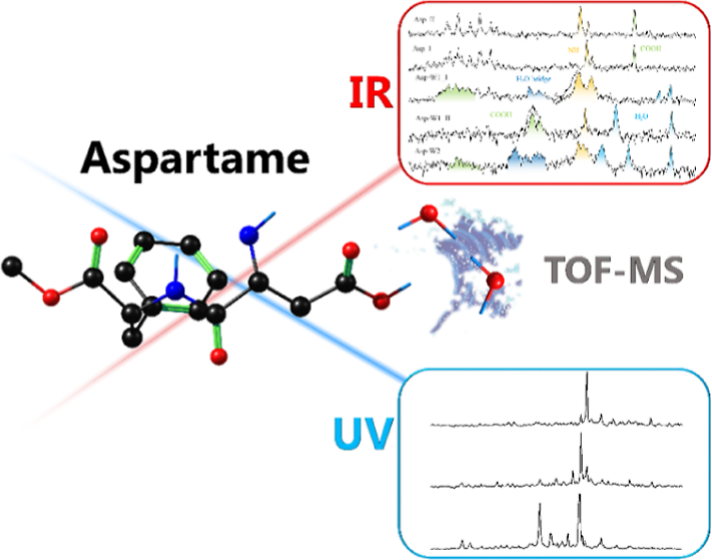

The popular sweetener, aspartame, is an agonist of the tongue’s sweet taste receptor. How water molecules affect its conformation or which aspartame atoms are more prone to interact with solvent are helpful questions to understand its activity in different environments. Here, the combination of IR–UV spectroscopic techniques with computational simulations has been successfully applied to characterize aspartame·water_0–2_ clusters, showing that the addition of water molecules simplifies the conformational panorama of aspartame, favoring the formation of folded structures by interaction with the polar part of the molecule.

## Introduction

**Scheme 1:**
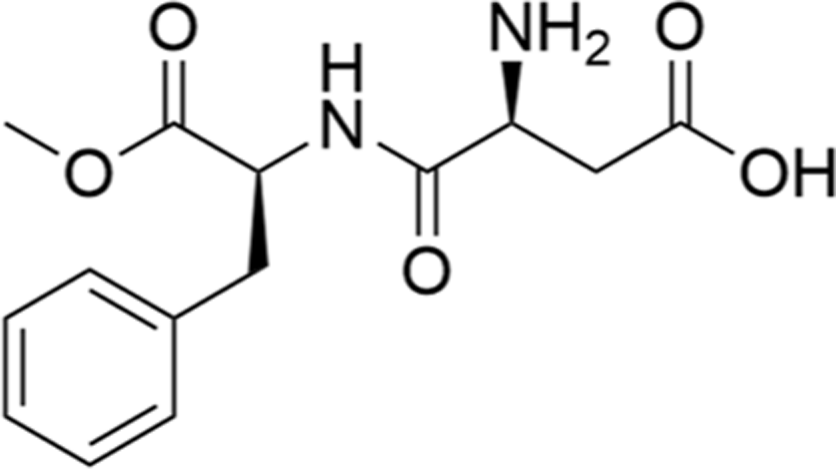
Aspartame (*N*-(l-α-Aspartyl)-l-phenylalanine, 1-Methyl Ester)

Aspartame is an artificial sweetener formed by the combination of two amino acids, aspartic acid and phenylalanine, and ended by a methyl ester bond (Scheme 1). Thus, aspartame mimics the structure of certain natural peptides. Its sweet flavor is due to its high affinity for taste receptors^1,2^ even at very low concentrations and, therefore, it is used in food industry to sweeten products with a small increase in their total calories. Nevertheless, World Health Organization’s (WHO’s) International Agency for Research on Cancer (IARC) has recently classified aspartame for the first time as a Group 2B agent, which is “possibly carcinogenic to humans”,^3^ making necessary continuous scrutiny and research regarding its impact on human health.

Determining the exact molecular mechanisms behind both beneficial and side effects of aspartame is a complicated task because it involves obtaining a precise knowledge of the interactions between the target molecule and many other receptors and proteins. Furthermore, all of these processes take place in the intra- (or inter-) cellular medium, whose main component is water. Therefore, it is necessary to understand not only the structure of aspartame but also the influence of water in its conformational preferences.

Many authors have reported a strong influence of the first water molecules on the modulation of the structure of biomolecules.^4−6^ Such studies gave rise to the concept of “biological water” to refer, e.g., to those few water molecules that get trapped in a receptor together with a ligand and that are necessary for the whole signaling process to start. However, extracting reliable experimental data on the true influence of those few water molecules on the structure of a biomolecule is not an easy task.

The combination of conformer-selective laser spectroscopy techniques and molecular beams, together with the faithful support of computational simulations, provides a solid methodology to unravel molecular aggregates. This is the approach used in most of the preceding studies on the influence of water molecules in the structure of biomolecules and peptides and has demonstrated an enormous potential to unravel the influence of the environment on molecular conformation.^7^

Actually, a reduced number of neutral amino acid·water_*n*_ aggregates have been previously studied with this or similar approaches,^8^ such as alanine·water_1–2_,^9^ tryptophan·water_0–3_,^10,11^ tryptophan·water_1–6_,^12^l-phenylalanine·water_0–3_ aggregates,^13,14^ or glycine·water_*n*_ (*n* = 0–9).^15−19^ Many of them aim to comprehend the conditions and the quantity of water molecules required to stabilize the zwitterionic form of the molecule, a task in which computational approaches can prove highly beneficial.^20−32^

Moreover, to study the effect of solvation on the peptides’ backbone, capped amino acids such as Ac-Phe-OMe·water_1–3_ clusters^33^ and phenylalanine derivatives, CH_3_–CO-Phe-NH_2_ and CH_3_–CO-Phe-NH–CH_3_,^34^ have been used.

Finally, several microsolvation studies carried out on systems that mimic a peptide bond, such as formamide,^35,36^*N*-phenylformamide,^37^*N*-benzyl formamide,^38^*trans*-formanilide,^39,40^ 2-phenyl acetamide,^41,42^ 2-pyridone,^18^ oxindole,^43^ or 3,4-dihydro-2(1H)-quinolinone,^43^ show cyclic structures mediated by water, with the C=O and N–H groups in cis conformation.

In the last years, important progress in the study of the conformation of small biomolecules in microsolvation conditions has been achieved using cryogenic ion spectroscopy techniques (see examples^44−49^). In those studies, the molecule of interest is monoprotonated or accompanied by a positively charged alkali metal, which enables trapping the species and further exploration using several spectroscopic techniques.

## Methods

### Experimental Section

The experimental setup used for this work^50,51^ was built around a time-of-flight mass spectrometer (TOF, Jordan) coupled to an in-house designed supersonic expansion chamber. Aspartame (Asp–Phe methyl ester, Tokyo Chemical Industries, >98%) was grounded with traces of graphite and rubbed on the surface of a solid graphite bar forming a thin layer. The graphite bar acts both as a sample holder and substrate for the sample ablation and is affixed to the flange of a pulsed valve (Jordan PSV) operated at 10 Hz with a backing pressure of 5 bar of neon. Light from a Nd/YAG laser (Minilite Continuum, 500 mJ/pulse) was used to desorb the sample synchronously with the opening of a pulsed 0.5 mm supersonic valve. Thus, the supersonic expansion created by the buffer gas picked and cooled the sample molecules. To produce hydrated complexes, the carrier gas was seeded with H_2_O or D_2_O upstream of the jet.

Once formed, the molecular beam was directed through a 3 mm skimmer (Beam Dynamics) to the differentially pumped ionization chamber of a TOF mass spectrometer, where it interacted with the spectroscopy lasers. The molecules and complexes were detected by measuring their one-color resonantly enhanced two-photon ionization (1c-R2PI) signal. A frequency doubled dye laser (FL2002, Lambda Physics, Coumarin 540A, 500 mJ/pulse) was used to produce excitation UV photons. The 1c-R2PI spectrum was obtained by monitoring the ion signal produced by the UV photons crossing the molecular beam while scanning the region of the π–π* electronic transition of the aromatic ring. To record conformer-specific, mass-resolved vibrational spectra of the molecules and complexes isolated in the molecular beam, we used a double resonance infrared ion depletion (IRID) spectroscopy scheme. The tunable IR photons were produced by a Nd/YAG pumped OPO/A system (LaserVision, 5 mJ/pulse). First, the UV laser was fixed in a probe frequency of the conformer of interest. Then, the IR laser beam interacted with the molecules 150 ns before the UV probe laser, producing a depletion in the ion signal whenever a vibrational transition was excited. By monitoring this ion depletion while scanning the IR region, we obtained the conformer-specific IRID spectrum.

### Computational Details

The conformational search of the modeled aggregates was performed using CREST software^52^ applying the semiempirical tight binding GFN2-xTB method^53^ for the calculations. Obtained structures were further optimized at the B3LYP-GD3BJ/def2-TZVP level as it is implemented in Gaussian 16 program.^54^ IR spectra were simulated by using the normal modes obtained within the harmonic approximation at the same level of theory. To take into account the anharmonicity of the vibrations and the deficiencies in the electronic structure of the method used during the spectral simulation, a scaling factor was applied to specific functional groups of aspartame. The scaling factors for each functional group were obtained through the analysis of the bare aspartame molecule and by comparison with other empirically determined scaling factors in different systems.^55^ In this way, correction factors of 0.964, 0.9605, and 0.963 were obtained for OH, NH, and CH vibrations, respectively. In order to simulate the experimental trace, the predicted frequencies were represented with a Lorentzian function of fwhm ≥5 cm^–1^ depending on the type and strength of the interaction and determined using the values reported in Table S1 of the Electronic Supporting Information (ESI).^56^ The resulting spectra were convolved with a Gaussian function (fwhm = 6 cm^–1^) to simulate the effect of the laser on the shape of the transition.

For each isomer and complex, free Gibbs energies were calculated at 0 and 298 K, including the zero-point energy correction. Thus, the relative energy presented in the figures is the difference between the Gibbs free energy of the denoted conformer and the most stable one.

These two temperatures were selected for the Gibbs energy calculation since the most stable structure at 0 K is typically observed in the molecular beam, although its vibrational temperature is estimated around 50–100 K.^57−60^ Nevertheless, some population, stabilized by entropic effects at room temperature (considered in Gibbs energy calculations at 298 K), could also become trapped in other local minima stable at higher temperatures, as shown in previous publications.^55,61−63^

## Results and Discussion

We add here to those previous studies using the above-mentioned methodology to characterize how the first two water molecules promote structural changes in aspartame. In principle, the most appealing solvation points should be the amino acid groups (Scheme 1). However, the first water molecules can perturb the original conformation of a peptide to create a more hydrophilic environment. Such conformational changes need to be mapped and taken into account to understand the interaction with the sites of action of a biomolecule in order to fully understand the molecular mechanisms behind its biological effect.

**Figure 1:**
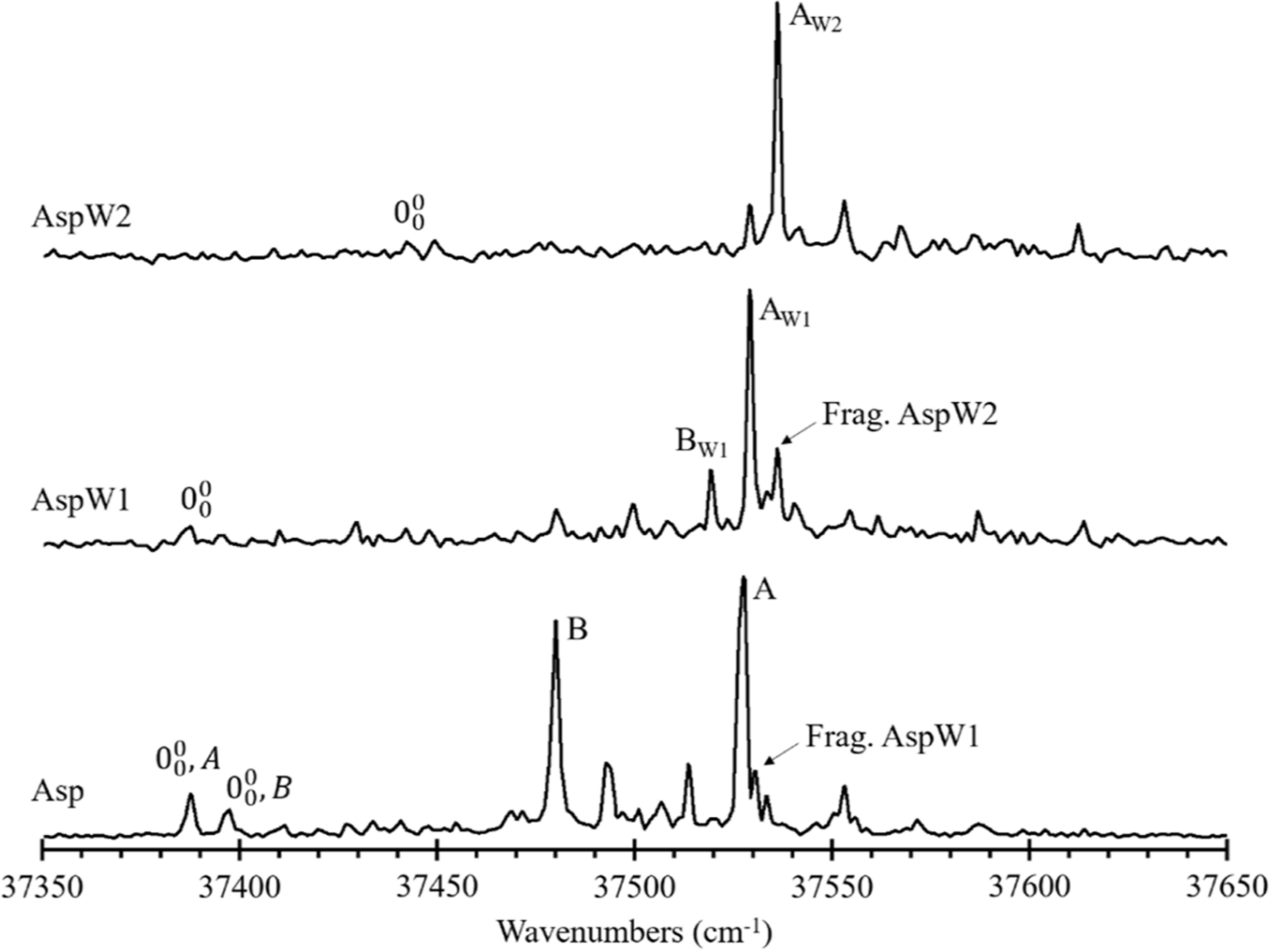
1c-R2PI spectra for (bottom panel) aspartame (Asp), (middle) monohydrated aspartame (AspW1), and (upper panel) doubly hydrated aspartame (AspW2). “Frag.” denotes the bands due to fragmentation from higher-order species.

Figure 1 collects the 1c-R2PI spectra of aspartame (lower spectrum) and its mono- (middle) and doubly hydrated complexes (upper spectrum).

Two conformers, labeled as A and B with 0_0_^0^ transitions at 37,387 and 37,397 cm^–1^, respectively, were observed in the 1c-R2PI spectrum of the aspartame molecule (Asp, lower panel). In the case of the monohydrated complex (AspW1, middle panel), also two conformers were detected, presenting 0_0_^0^ transitions at 37,529 and 37,519 cm^–1^, respectively. Conversely, only one conformer of the dihydrated complex (AspW2: upper panel of Figure 1), labeled as A_W2_, was found with 0_0_^0^ transition at 37,442 cm^–1^.

**Figure 2:**
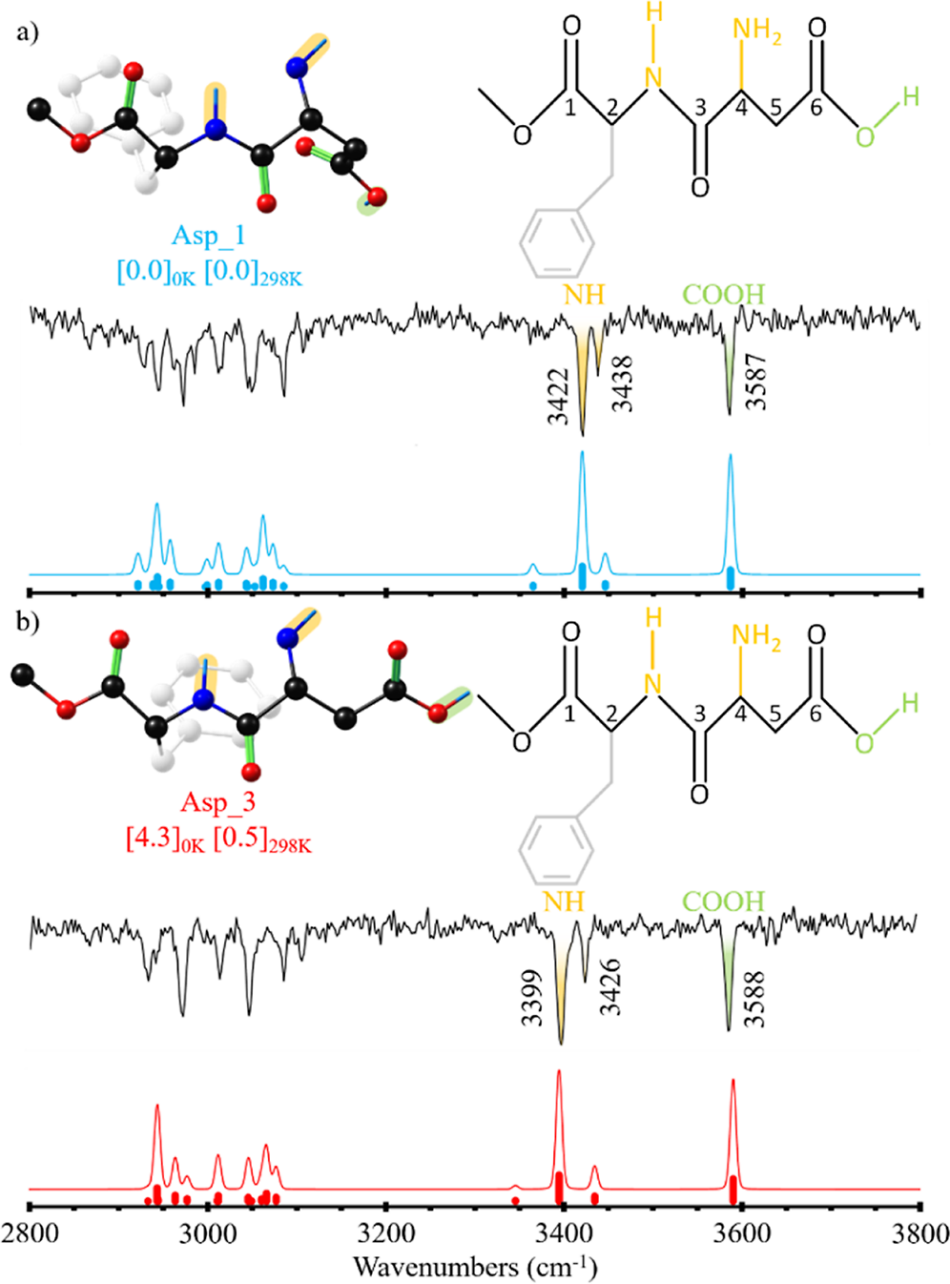
Comparison between the experimental IRID spectra obtained probing bands A (a) and B (b) in the 1c-R2PI spectrum of aspartame and the simulations for Asp_1 (a) and Asp_3 (b) conformers. Scaling factors of 0.964, 0.9605, and 0.963 were used for OH, NH, and CH stretching vibrations, respectively, to account for anharmonicity. The numbers in brackets correspond to the relative stability in kJ/mol, calculated as the difference between the Gibbs free-energy values of the denoted conformer and the most stable one at 0 and 298 K.

**Figure 3:**
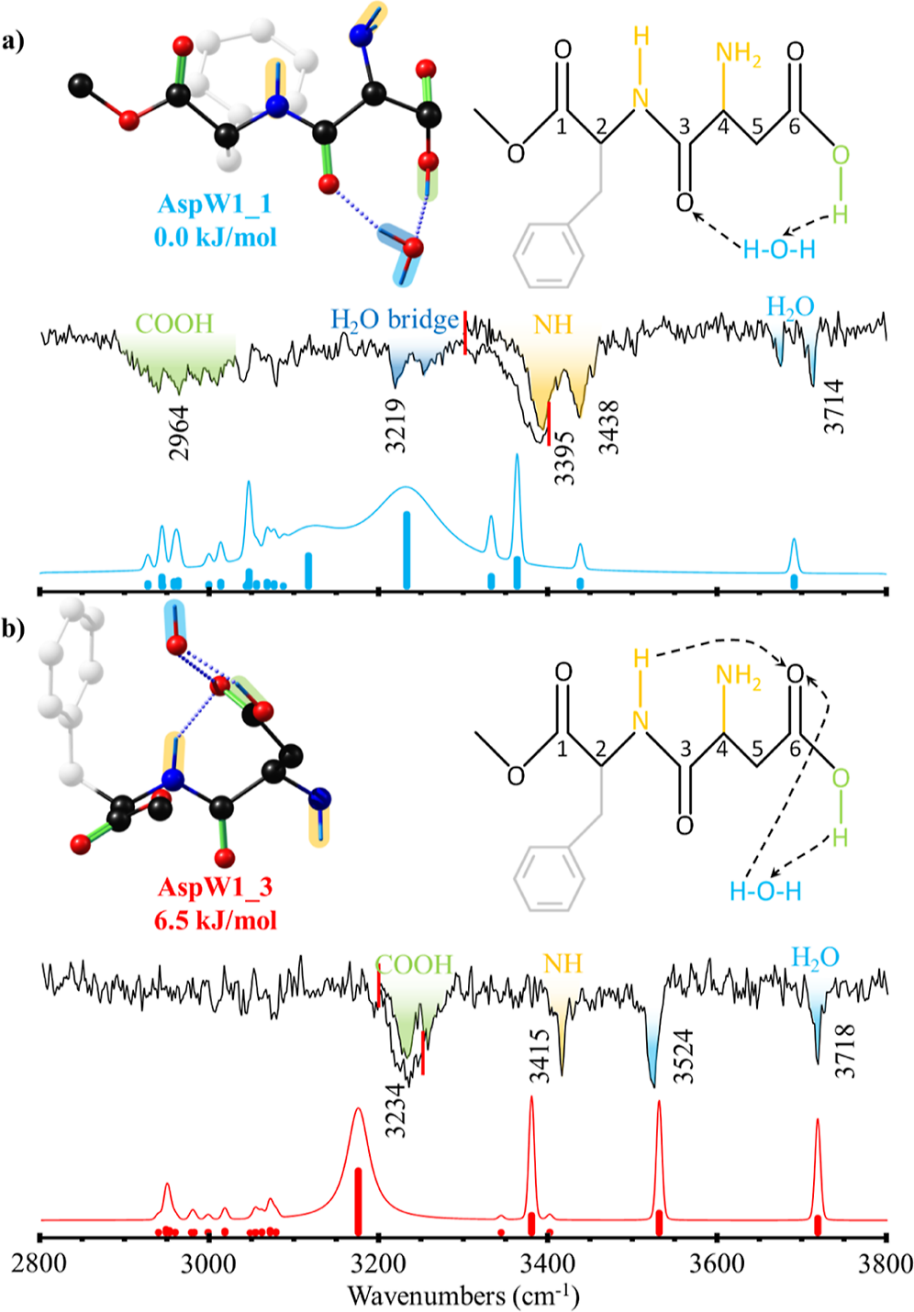
Comparison between the experimental IRID spectra obtained the A_W1_ (a) and B_W1_ (b) bands in the 1c-R2PI spectrum of the monohydrate in Figure 1 and the computational simulation for AspW1_1 (a) and AspW1_3 (b) conformers. Scaling factors of 0.964, 0.9605, and 0.963 were used for OH, NH, and CH stretching vibrations, respectively, to account for anharmonicity. The numbers in brackets correspond to the relative stability in kJ/mol, calculated as the difference between the Gibbs free-energy values of the denoted conformer and the most stable one at 0 and 298 K, respectively. Vertical red lines in the experimental trace of the B_W1_ conformer mark the starting and ending point of the two different scans used to obtain the experimental data.

**Figure 4:**
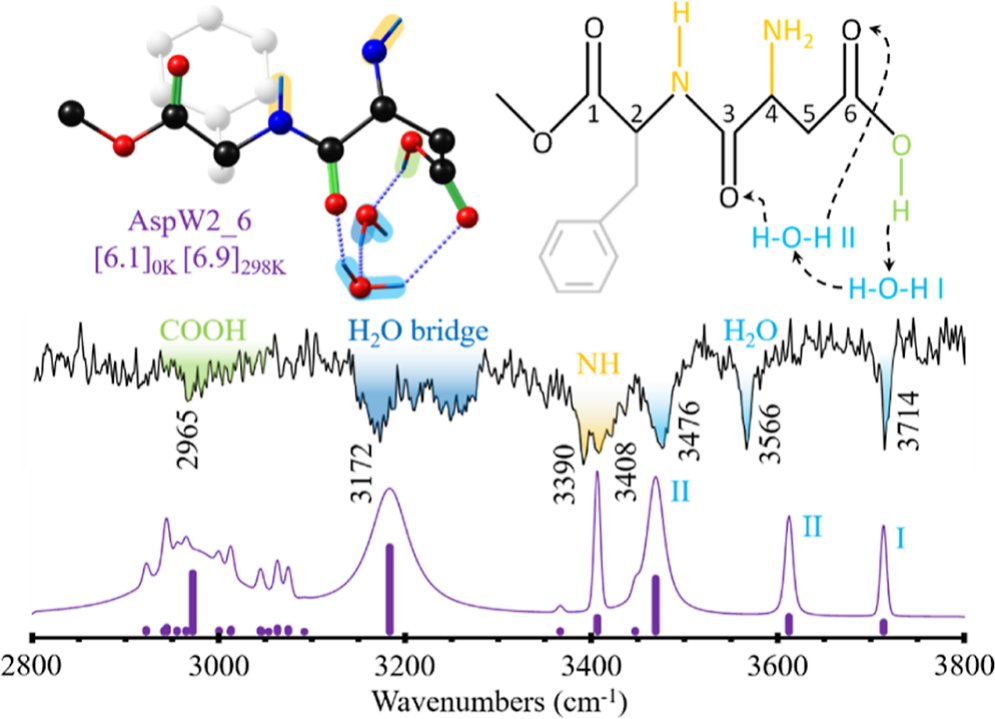
Comparison between the experimental IRID spectrum of A_W2_ and the simulation for the AspW2_6 conformer. Scaling factors of 0.964, 0.9605, and 0.963 for OH, NH, and CH, respectively, were used to account for anharmonicity. The numbers in brackets correspond to the relative stability in kJ/mol, calculated as the difference between the Gibbs free-energy values of the denoted conformer and the most stable one at 0 and 298 K.

The presence of two conformers in the spectrum of the monomer was further confirmed by using IR/UV hole burning (Figure S1 of the ESI). To test that no isomer was missing, all of the discrete transitions in the R2PI spectra were probed in IRID experiments, obtaining the two traces in Figure 2 for the monomer. Two different IRID spectra were found for the monohydrated (Figure 3) and a single isomer was found for the dihydrated (Figure 4).

As can be observed in Figure 1, there is a simplification in the number of electronic transitions as water molecules are added, together with a significant shift in the origin band of the AspW2 aggregate, pointing to an increase in the interaction strength of the aggregate upon excitation.

After isolating the IR spectrum corresponding to each observed conformation, these were compared with the theoretical traces simulated using the structures obtained in the conformational search carried out at the B3LYP-GD3BJ/def2-TZVP calculation level.

Figure 2 shows the comparison between the IRID spectra obtained for Asp and the simulations from the computed conformations that fit better the experimental data; i.e., Asp_1 and Asp_3, respectively.

Moreover, Figure S2 (ESI) shows the structures of the six most stable species (at 0 K) resulting from the exploration of the conformational space, together with their relative stability at 0 and 298 K. Furthermore, a collection of 16 calculated conformers for each aggregate is depicted in Figure S11 to illustrate the kind of structures found during the exploration of the conformational landscape, which in some cases include more than a hundred structures.

The comparison between the computed and experimental IR spectra may be found in Figure S3. The absence of conformer 2 despite being the second most stable structure may be due to its connection with the global minimum (conformer 1) by the rotation through the C4–C5 bond (see Figure 2 for carbon atom labels), which very likely presents a very low potential energy barrier, and therefore, it may be overcome during the cooling process.

As can be seen in Figure 2, the computational predictions reproduce reasonably well the experimental bands for both (a) and (b) spectra. The most stable structure, Asp_1, well reproduces all of the OH and NH stretching bands in trace (a). In the case of spectrum (b), the best match is provided by the third most stable structure. Despite Asp_3 being higher in electronic energy (see energy values in brackets in Figure 2), the conformation is stabilized by entropic effects as its energy at 298 K is closer to Asp_1.

Comparison with the computational predictions led us to assign the most energetic absorption to the O–H stretching mode (3587 and 3588 cm^–1^ in conformer A and B, respectively), in good agreement with previously reported benzoic acid^64^ and tyrosine^65^ experimental spectra, under similar experimental conditions. The band corresponding to the antisymmetric stretching mode of the –NH_2_ group lies above 3425 cm^–1^. The N–H stretching mode of the amide fragment is clear around 3400 cm^–1^, whereas the symmetric stretching mode from –NH_2_ is hard to identify in the spectra, as in tyrosine^65,66^ or other amine-containing molecules, such as aniline^67,68^ or tryptophan.^69^ Nevertheless, the symmetric stretching mode is theoretically predicted to be around 3350 cm^–1^ in both assigned conformers.

Comparison between the two assigned structures (Asp_1 and Asp_3) highlights that the main difference between them is the N–H···O=C hydrogen bond formed in Asp_1 (Figure 2a) that forces the carboxylic acid terminal to fold toward the backbone of the peptide (Figure 2b). This N–H···O interaction in Asp_1 surely produces the displacement to higher wavenumbers of –NH_2_ antisymmetric stretching in this conformer.

Figure 3 shows the experimental IRID spectra obtained for the two isomers of the monohydrated complex of Asp, compared to the best fit provided by the theoretical simulations. A summary of the structures found during the exploration of the conformational space of AspW1 and the corresponding comparison between computed and experimental IR spectra can be found in Figures S4 and S5, respectively.

Interestingly, the spectrum shown in Figure 3a presents two bands around 3700 cm^–1^ that were not reproduced by the theoretical simulation of any A_W1_ structure (see Figure S5). Transitions in this spectral region must arise from the stretching vibration of the water molecule’s free hydroxyl group (O_W_H). This was further demonstrated experimentally thanks to isotopic substitution carried out with deuterated water, where the analogous bands appeared in the 2350–2700 cm^–1^ range (see Figure S6). As the rest of the spectrum is in coherence with the number of bands corresponding to a single insertion of a water molecule in Asp, the observation of two vibrations in the region of free O_W_H vibration points to the contribution of two different isomers to the spectrum with very similar structures, maybe differing only slightly on the position of the water molecule. Furthermore, it was not possible to find a structure whose predicted spectrum could reproduce the position of the band at 3675 cm^–1^.

Following this hypothesis and due to the good agreement of the theoretical spectrum with the rest of the bands, this spectrum was assigned to the Asp_1 structure. In this aggregate, water inserts between the carboxylic OH and the oxygen atom of the amide bond of aspartame creating an eight-membered cyclic hydrogen bond network.

The comparison between experimental and simulated IR spectra for the B_W1_ conformer (Figure 3) shows good agreement with the predicted spectrum for the third most stable calculated conformer: Asp_3. In this computed structure, water inserts in the carboxylic acid group of Asp, acting simultaneously as a proton donor and acceptor, forming a cyclic hydrogen bond network. Moreover, the structure Asp_3 well reproduces the two water bands at 3718 and 3524 cm^–1^ (see Figure S7 for the experimental assignment of these bands with isotopic substitution). The band at 3718 cm^–1^ corresponds to the stretching vibration of the free OH, whereas the one at 3524 cm^–1^ is assigned to the stretch of the water’s OH that participates in the hydrogen bond with the carboxylic acid. A similar displacement to lower frequencies of the “bridge” hydrogen was also observed in the aggregates of benzoic acid^64^ and 3-indole propionic acid^70^ with one water molecule.

In both A_W1_ and B_W1_ conformers, the carboxylic O–H stretching vibration experiences a substantial red shift due to the formation of a strong hydrogen bond with the water molecule. In the case of A_W1_, the stretch of this OH_acid_ appears at 3252 cm^–1^ and at 3234 cm^–1^ in B_W1_. Besides, due to strong anharmonic effects,^71,72^ these bands appear as broad absorptions.

In addition to these interactions with water, both conformers show an additional intramolecular hydrogen bond. In the case of AspW1_1, this interaction is between – NH_2_ and acidic C=O, as in the case of Asp_1. Additionally, there is an intramolecular hydrogen bond in AspW1_3 between the – NH–H and the C=O of the acidic group, stabilizing the folded conformation of the backbone through an intramolecular C7 hydrogen bond, which is characteristic of the γ-turns in protein backbones.

Figure 4 shows a comparison between the IRID spectrum obtained for the doubly hydrated complex, A_W2_, and the simulations built by using the computed structures.

The exhaustive conformational exploration led to multiple, very stable local minima (see a summary in Figures S8, S9, and S11). Interestingly, there is a poor correlation between the simulated IR spectra of the first most stable isomers and the experimental one, especially in the higher wavenumber side of the spectrum, a region which is usually well predicted by the computational methods. The most stable structure whose predicted spectrum is able to reproduce the experimental results is isomer Asp_6, which is ca. 6 kJ/mol above the global minimum. Taking into account the size of the system, this energy difference is well within the computation error and may be pointing to a problem of this functional when trying to predict the relative stability of the species of the dihydrated aggregate. In AspW2_6, the second water molecule also inserts in the acid group of aspartame, extending the hydrogen bond network formed in AspW1_3. The free OH stretching vibration of water molecule I appears at 3714 cm^–1^, while those of the OH taking part in hydrogen bonds appear at 3566 and 3476 cm^–1^. This second water molecule (II) acts as a bridge, closing the cooperative hydrogen bond network with both amidic and carboxylic C=O groups. Nonetheless, due to an intense cooperative and anharmonic effect, the acid OH vibration is strongly shifted to lower frequencies, and it is predicted to appear as a very broad absorption around 2950 cm^–1^. A similar situation is observed for the broad band around 3172 cm^–1^, since this one is predicted to be the OH stretch of the water in bridged position and its combination bands, as observed in benzoic acid·water_2_ and 3-indole propionic acid·water_2_ aggregates.^64,70^

The comparison between all of the obtained IRID spectra (see Figure S10) shows a clear shift of the assigned OH_acid_ vibration bands to lower frequencies as the number of inserted water molecules increases. Thus, despite the numerous solvation positions available due to the presence of multiple polar groups in aspartame, the carboxylic acid combined with the flexibility of the peptide backbone creates a hydrophilic binding site that becomes the preferred solvation site for the first two water molecules. Such preference was already described for isolated tyrosine, where the hydrogen bond is formed between the carboxylic – OH and – NH_2_.^65^ Actually, the reduction of the acid OH stretching frequency with the insertion of water molecules, together with the strong cooperative and anharmonic hydrogen bond interactions observed in the assigned structures, may suggest that the successive addition of more water molecules would promote a proton transfer reaction resulting in zwitterion formation.^13^ On the other hand, aspartame’s NH stretching vibration bands are also broadened when – NH_2_ or amidic – NH– interact with the solvent, as in A_W1_ and A_W2_ species, although their position is not so dramatically modified.

## Conclusions

The analysis of the experimentally assigned aspartame conformations shows that the progressive addition of water molecules seems to simplify the conformational panorama and its interaction preferences. The former can be related to the creation of a first solvation shell, a phenomenon also observed in studies of l-phenylalanine·water_0–3_ aggregates,^13^ capped amino acids as Ac-Phe-OMe,^33^ or phenylalanine derivatives in microsolvation environments.^34^

**Figure 5:**
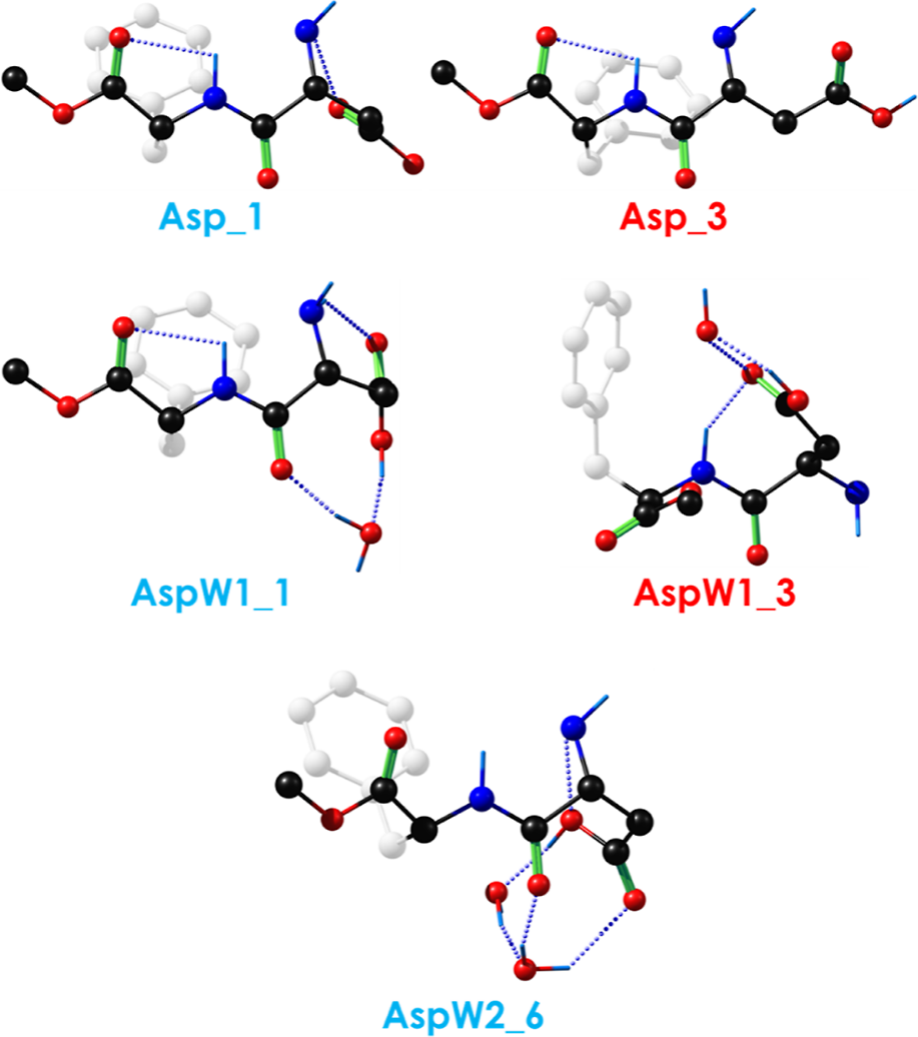
Calculated structures assigned to the conformers detected in the experiments.

As can be seen in Figures 2 or 5, where all of the assigned structures to the experimentally detected conformers are collected, for the case of isolated Asp, both observed stable conformations present the peptide in an extended or semifolded conformation, with the characteristic intramolecular C5 hydrogen bond typically observed in β-sheet arrangements. Although the – COOH terminal group is free, we observe no completely folded structures due to the formation of medium to high strength intramolecular hydrogen bonds involving the acid terminal.^73^ However, this trend changes upon microsolvation.

Two different structures were assigned for the monohydrated complex (see Figures 3 and 5). In both, the interaction with water folds the aspartame structure. Previous studies of peptides in the gas phase show two tendencies: the peptide’s structure can resist microsolvation or the insertion of one water molecule induces a change in the conformation.^10,13,15^ In this case, inclusion of water molecules seems to favor the folded structure of the sweetener. Regarding the question of whether extended aspartame conformations interact less effectively with water molecules or if these water molecules, in fact, force the molecule to fold, the latter becomes more plausible after comparing our present results to previous studies. For example, in tryptophan·water_0–3_^11^ or capped phenylalanine derivatives, e.g., CH_3_–CO-Phe-NH_2_ and CH_3_–CO-Phe-NH–CH_3_,^34^ the insertion of water molecules produces important changes in the conformational preferences of the amino acid. Likewise, structural changes were observed in AspW2_6 (Figures 4 and 5), where the insertion of a second water molecule forces the acid terminal of the aspartic acid residue to adopt a semifolded conformation, mainly driven by the formation of a strong and cooperative cyclic hydrogen bond network between the COOH group and the water molecules.

The participation of acid groups is something expected due to the polar nature of the involved bonds and previous results in glycine·water_1_,^15^ where the – COOH fragment is the preferred interaction site for water, which forms cyclic hydrogen bond networks. Thus, water ignores the ester part of the aspartame molecule, highlighting the difference between the hydrophilic and hydrophobic parts of the aspartame molecule. This difference is important since docking^1^ and molecular dynamics^2^ simulations on aspartame binding in human homology models of T1R2 units as part of the sweet taste receptor show a clear differentiation in the anchoring groups, the “polar part” of aspartame being the main point for its accommodation on the T1R2 active site.^1,2^

The above-mentioned references show aspartame in its zwitterionic form, while such a state is not observed under our experimental conditions or in the calculations. It is clear in the IRID spectra that the carboxylic–OH stretching band is displaced toward low frequencies but still detected. Thus, the water molecules seem to favor a conformational change to the folded aspartame and a reduction in the number of species detected in the beam before stabilization of the zwitterion. Nevertheless, this poses the question of how many water molecules are required to stabilize the zwitterionic form in aspartame. Delving into more complex microsolvated aggregates and providing further experimental and theoretical studies would contribute to place one more piece in the complex puzzle of understanding biological activities of simple biomolecules and the role played by water.

## Supporting Information Available

Additional spectra of the species studied in this work including deuterated aggregates, and figures with further computed structures and energies at zero and room temperature (PDF)
