# Supplementary material for: Aspartame and Its Microhydrated Aggregates Revealed by Laser Spectroscopy: Water–Sweetener Interactions in the Gas Phase
Source: J Phys Chem A. 2024 Aug 2;128(32):6714–21. doi: 10.1021/acs.jpca.4c04315 (PMC11331506; doi:10.1021/acs.jpca.4c04315)
Supplement: Supplementary file 1 — jp4c04315_si_001.pdf [file jp4c04315_si_001.pdf]

Supplementary Information for

Aspartame and its Micro-Hydrated Aggregates Revealed by Laser Spectroscopy: Water-Sweetener Interactions in Gas-Phase

Paul Pinillos,[a,†] *Ander Camiruaga,*[b,†] *Fernando Torres-Hernández,*[a] *Pierre Çarçabal,*[b] *Imanol Usabiaga,*[a] *José A. Fernández*[a,]* *and Rodrigo Martínez.*[c,]*

[a] Dep. of Physical Chemistry, Fac. of Science and Technology, University of the Basque Country (UPV/EHU), Bº Sarriena S/N, Leioa 48940, Spain.

[b] Institut des Sciences Moléculaires d'Orsay (ISMO), Université Paris Saclay, CNRS, 91405 Orsay, France.

[c]Dep. of Chemistry, Fac. of Science and Technology, Univ. of La Rioja, Madre de Dios 53, 26006, Logroño, Spain.

[†]These two authors contributed equally to this work

Corresponding authors: José A. Fernández josea.fernandez@ehu.es , Rodrigo Martínez rodrigo.martinez@unirioja.es

TABLE OF CONTENTS

Table S1 FWHM values used for the simulation of the theoretical spectra. **Page S4.**

Figure S1.1c-R2PI (black traces) and IR-UV hole burning (blue and red traces) spectra for A (lower panel) and B (upper panel) conformers of aspartame. Conformer A IR-UV spectrum was recorded probing the transition at 3400 cm-1, whereas that of Conformer B was recorded probing the transition at 3420 cm-1. **Page S5.**

Figure S2. Most stable B3LYP/def2-VTZ computed conformers and relative energies (in kJ/mol) at 0 and 298 K of aspartame in brackets, calculated as the difference between the Gibs free energy values of the denoted conformer and the most stable at 0 and 298 K, respectively. The most important interaction in each conformer is labelled in coloured rectangles. **Page S6.**

Figure S3. Experimental (black) and B3LYP/def2-VTZ (coloured) IR spectra for aspartame molecule. Scaling factors 0.964, 0.9605, and 0.963 were used for OH, NH, and CH stretching vibrations, respectively, to account for anharmonicity. A and B labels indicate the corresponding experimental conformer. **Page S7.**

Figure S4 Most stable B3LYP/def2-VTZ computed conformers and relative energies (in kJ/mol) at 0 and 298 K of aspartame·water$_1$ aggregate in brackets, calculated as the difference between the Gibs free energy values of the denoted conformer and the most stable at 0 and 298 K, respectively. The most important interaction in each conformer is labelled in coloured rectangles. **Page S8.**

Figure S5. Experimental (black) and B3LYP/def2-VTZ (coloured) IR spectra for aspartame·water$_1$ aggregate. Scaling factors 0.964, 0.9605, and 0.963 were used for OH, NH, and CH stretching vibrations, respectively, to account for anharmonicity. A_{W1} and B_{W1} labels indicate the experimental conformer, whereas the vertical red lines in the experimental traces mark the starting and ending point of the two different scans used to obtain the experimental data. **Page S9.**

Figure S6. Experimental IRID spectrum for A_{D1} and computational simulation for AspD1_1. Scaling factors 0.964, 0.9605, and 0.963 were used for OH, NH, and CH stretching vibrations, respectively, to account for anharmonicity. **Page S10.**

Figure S7. Experimental IRID spectrum for B_{D1} and computational simulation for AspD1_3. Scaling factors 0.964, 0.9605, and 0.963 were used for OH, NH, and CH stretching vibrations, respectively, to account for anharmonicity. **Page S11.**

Figure S8 Most stable B3LYP/def2-VTZ computed conformers and relative energies in (kJ/mol), at 0 and 298 K of aspartame·water$_2$ aggregate, calculated as the difference between the Gibs free energy values of the denoted conformer and the most stable at 0 and 298 K, respectively. The most important interaction in each conformer is labelled in coloured rectangles. **Page S12.**

Figure S9. Experimental (black) and B3LYP/def2-VTZ (coloured) IR spectra for A_{W2} aggregate. Scaling factors 0.964, 0.9605, and 0.963 were used for OH, NH, and CH stretching vibrations, respectively, to account for anharmonicity. **Page S13.**

Figure S10. Summary of the experimental IRID spectra for aspartame·water$_{0\text{-}2}$ aggregates. The vertical red lines in the experimental traces mark the starting and ending point of different scans used to obtain the experimental data. **Page S14.**

Figure S11. Some example structures resulting from the conformational search. For Asp (a), Asp_W1 (b), and Asp_W2 aggregates. **Page S15.**

Table S1 FWHM values used for the simulation of the theoretical spectra. *

Spectral region (cm^{-1})	>3550	<3550	<3500	
FWHM for OH	5	10	20	
Spectral region (cm^{-1})	>3400	<3400	<3350	<3300
FWHM for NH	5	10	20	25

* Usabiaga, I. et al. Physical Chemistry Chemical Physics 2020, **22** (27), 15759-15768.

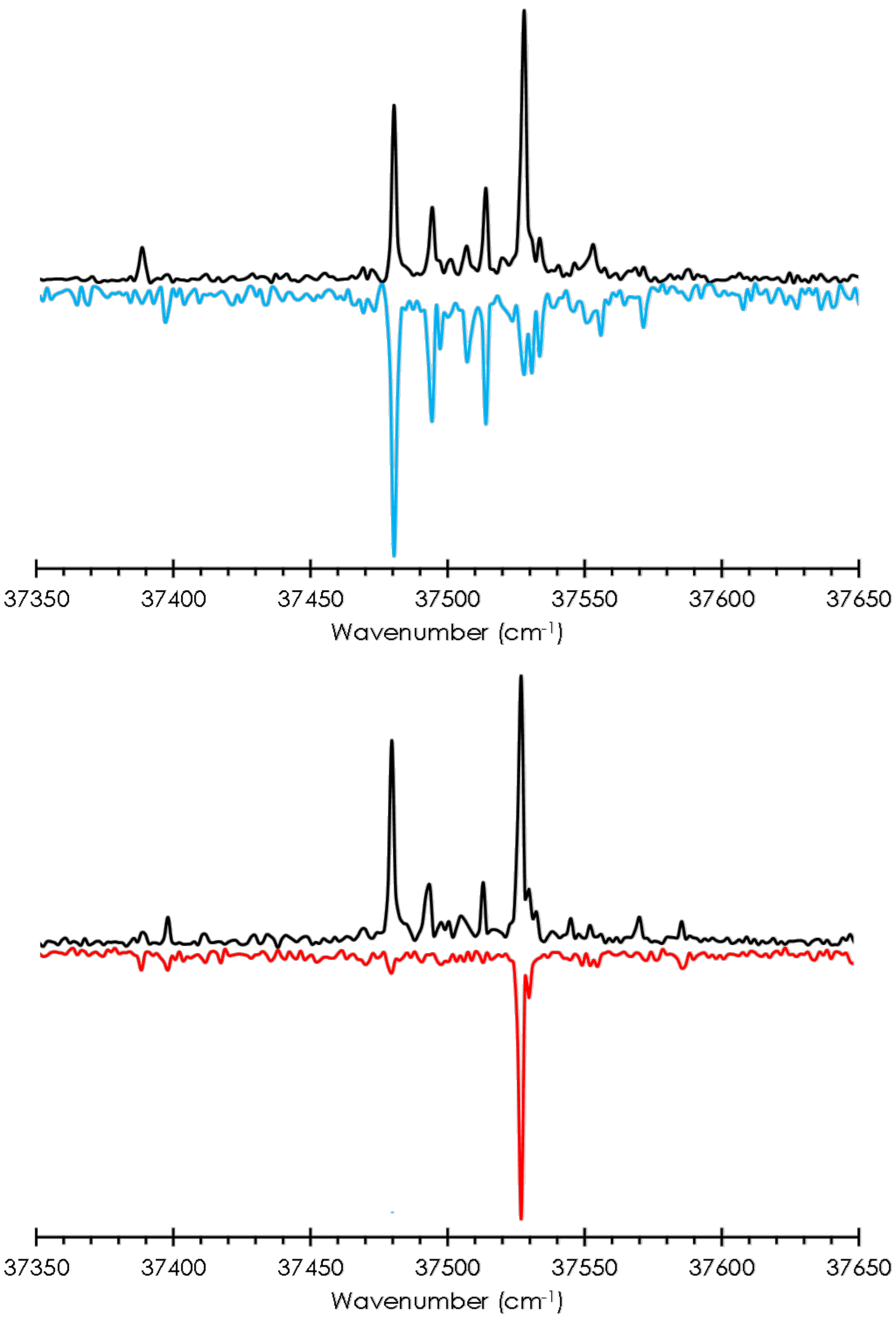

Figure S1.1c-R2PI (black traces) and IR-UV hole burning (blue and red traces) spectra for A (lower panel) and B (upper panel) conformers of aspartame. Conformer A IR-UV spectrum was recorded probing the transition at 3400 cm-1, whereas that of Conformer B was recorded probing the transition at 3420 cm-1.

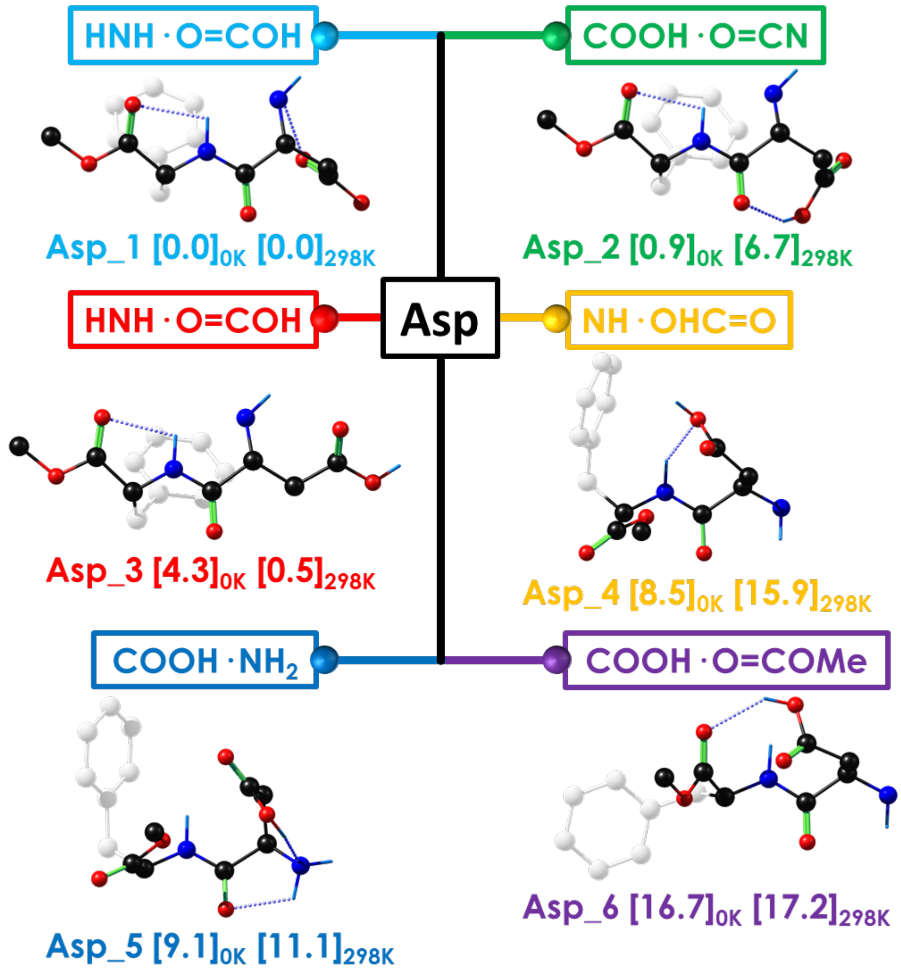

Figure S2. Most stable B3LYP/def2-VTZ computed conformers and relative energies (in kJ/mol) at 0 and 298 K of aspartame in brackets, calculated as the difference between the Gibs free energy values of the denoted conformer and the most stable at 0 and 298 K, respectively. The most important interaction in each conformer is labelled in coloured rectangles.

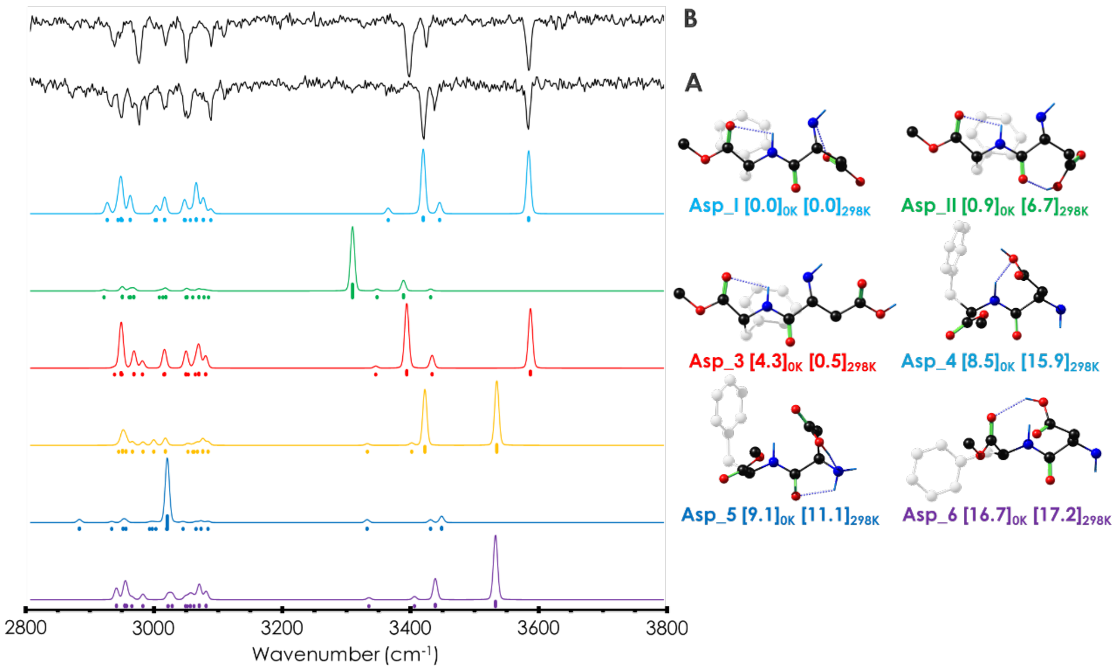

Figure S3. Experimental (black) and B3LYP/def2-VTZ (coloured) IR spectra for aspartame molecule. Scaling factors 0.964, 0.9605, and 0.963 were used for OH, NH, and CH stretching vibrations, respectively, to account for anharmonicity. A and B labels indicate the corresponding experimental conformer.

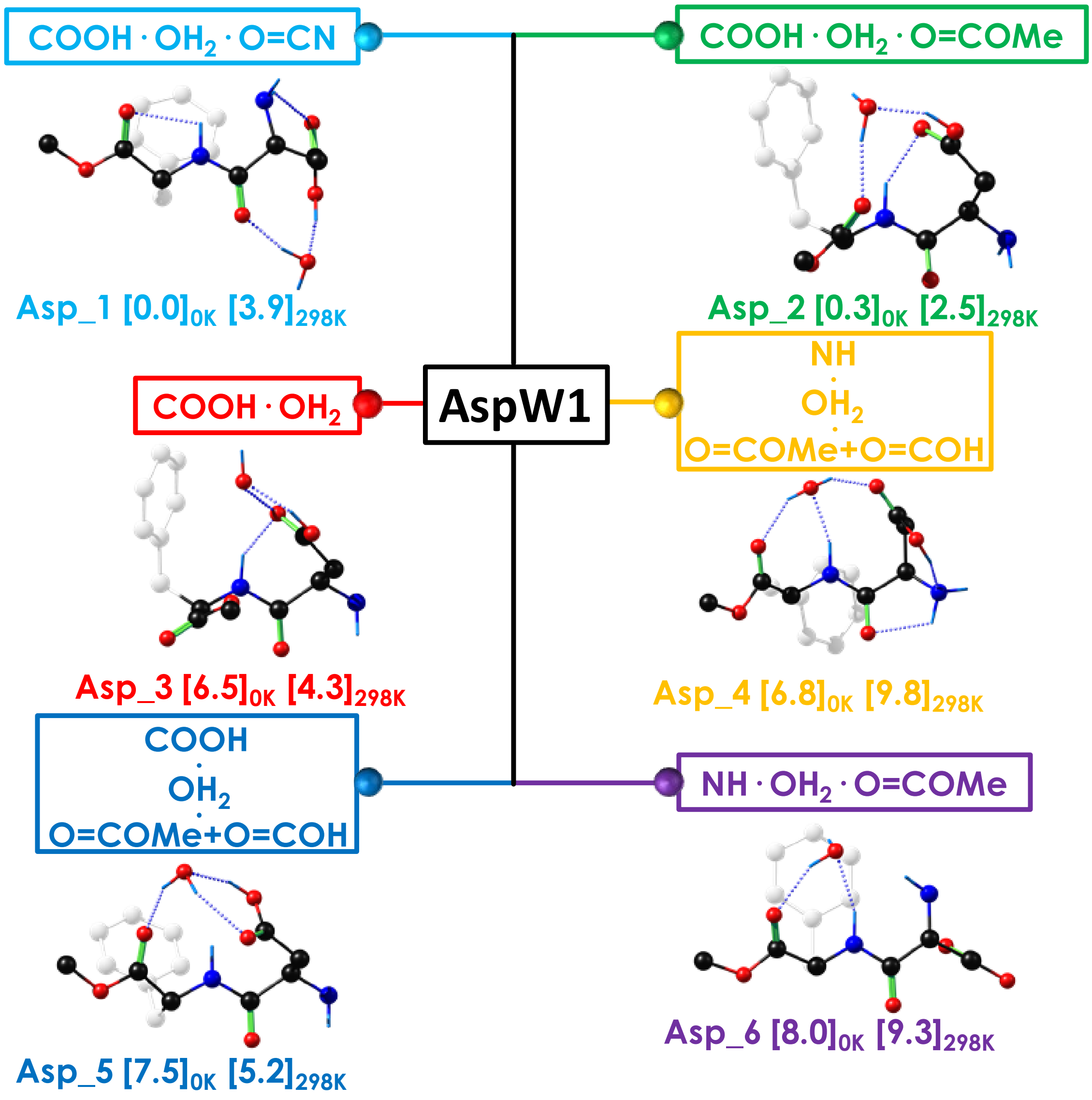

Figure S4. Most stable B3LYP/def2-VTZ computed conformers and relative energies (in kJ/mol) at 0 and 298 K of aspartame·water$_1$ aggregate in brackets, calculated as the difference between the Gibs free energy values of the denoted conformer and the most stable at 0 and 298 K, respectively. The most important interaction in each conformer is labelled in coloured rectangles.

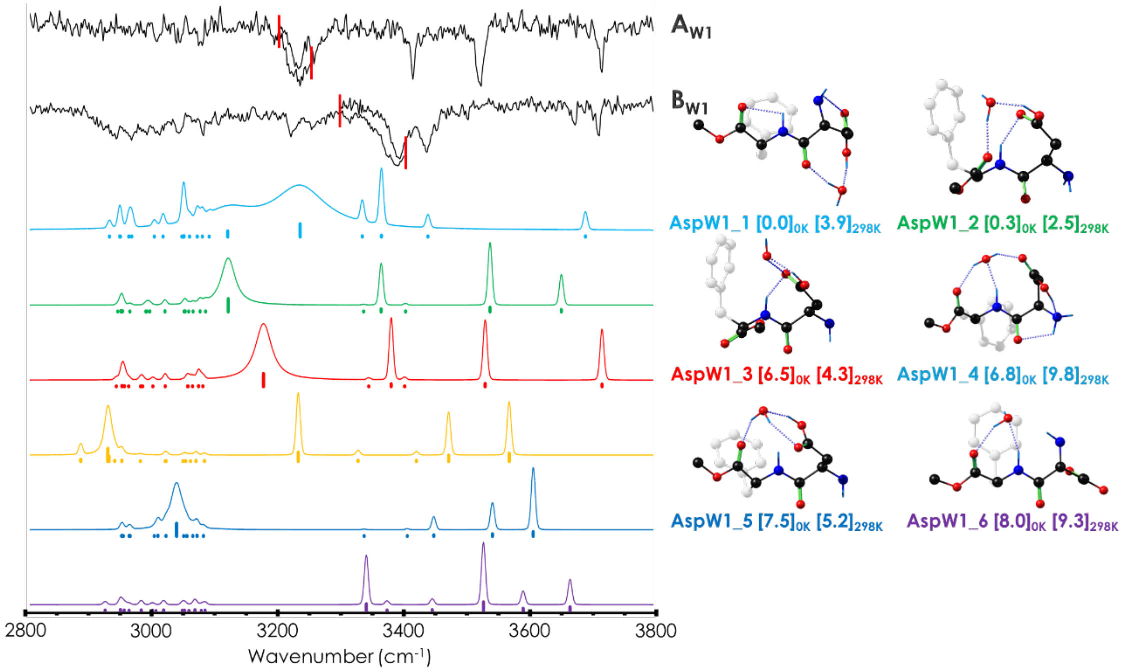

Figure S5. Experimental (black) and B3LYP/def2-VTZ (coloured) IR spectra for aspartame·water$_1$ aggregate. Scaling factors 0.964, 0.9605, and 0.963 were used for OH, NH, and CH stretching vibrations, respectively, to account for anharmonicity. A_{w1} and B_{w1} labels indicate the experimental conformer, whereas the vertical red lines in the experimental traces mark the starting and ending point of the two different scans used to obtain the experimental data.

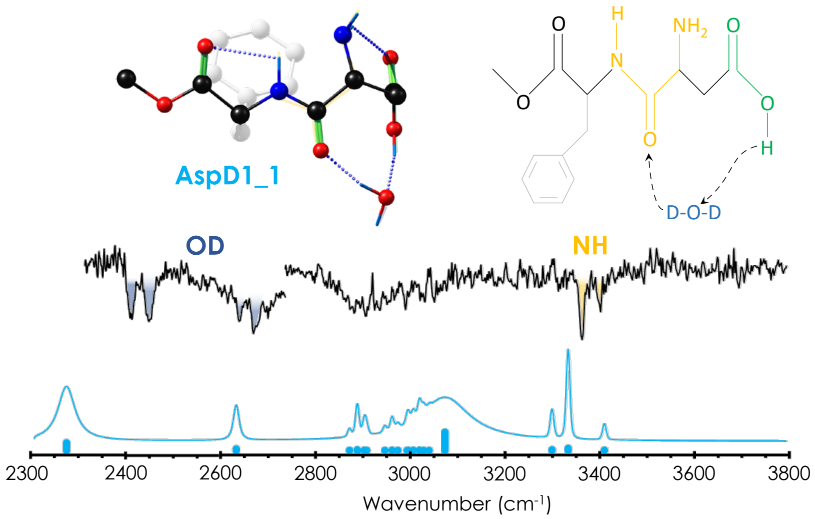

Figure S6. Experimental IRID spectrum for A_{D1} and computational simulation for AspD1_1. Scaling factors 0.964, 0.9605, and 0.963 were used for OH, NH, and CH stretching vibrations, respectively, to account for anharmonicity.

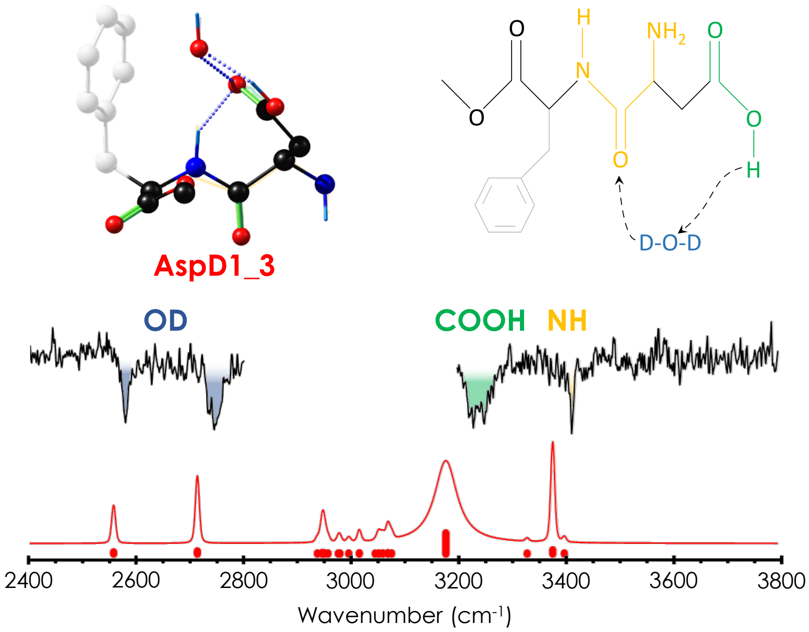

Figure S7. Experimental IRID spectrum for B_{D1} and computational simulation for AspD1_3. Scaling factors 0.964, 0.9605, and 0.963 were used for OH, NH, and CH stretching vibrations, respectively, to account for anharmonicity.

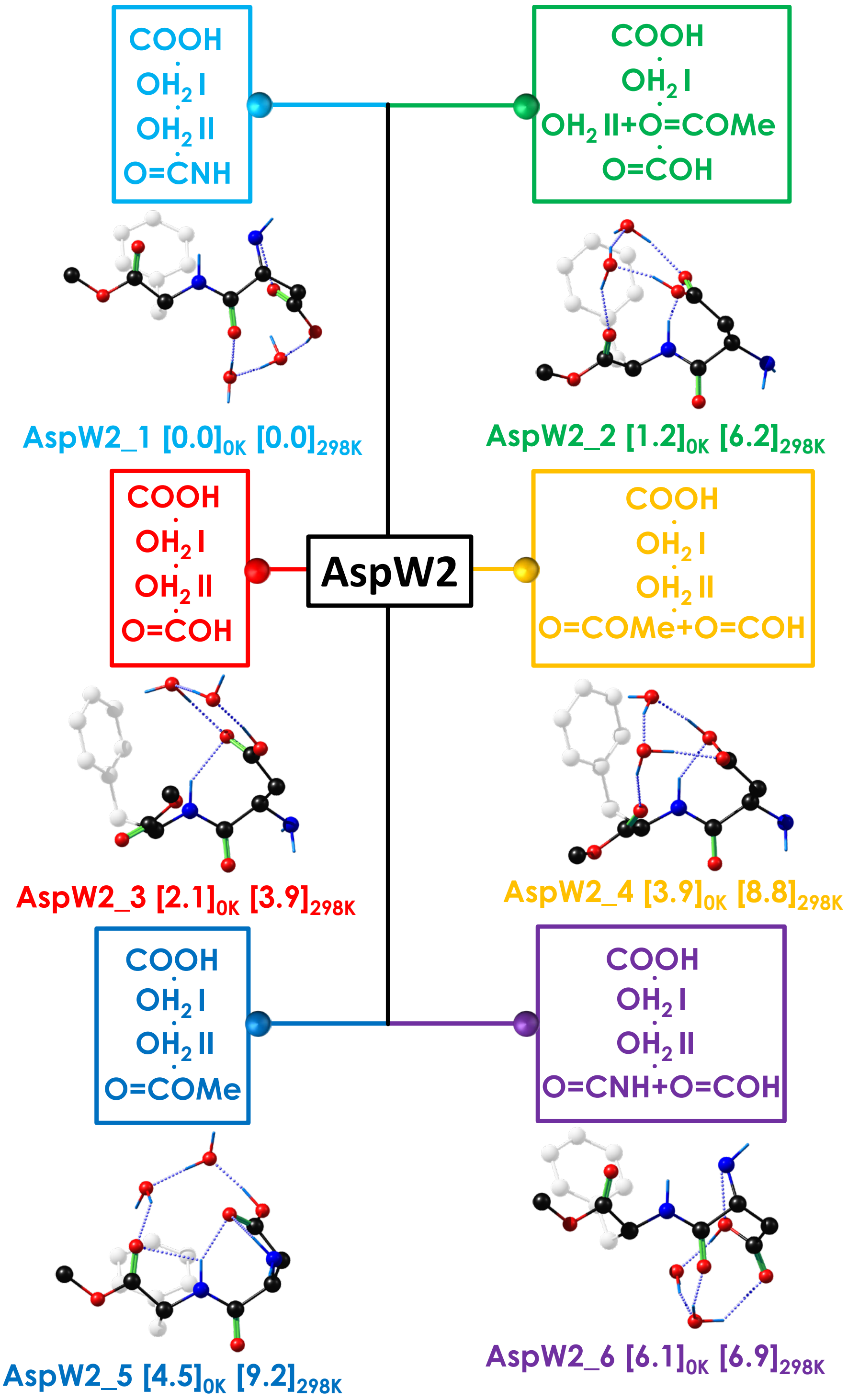

Figure S8. Most stable B3LYP/def2-VTZ computed conformers and relative energies in (kJ/mol), at 0 and 298 K of aspartame·water$_2$ aggregate, calculated as the difference between the Gibs free energy values of the denoted conformer and the most stable at 0 and 298 K, respectively. The most important interaction in each conformer is labelled in coloured rectangles.

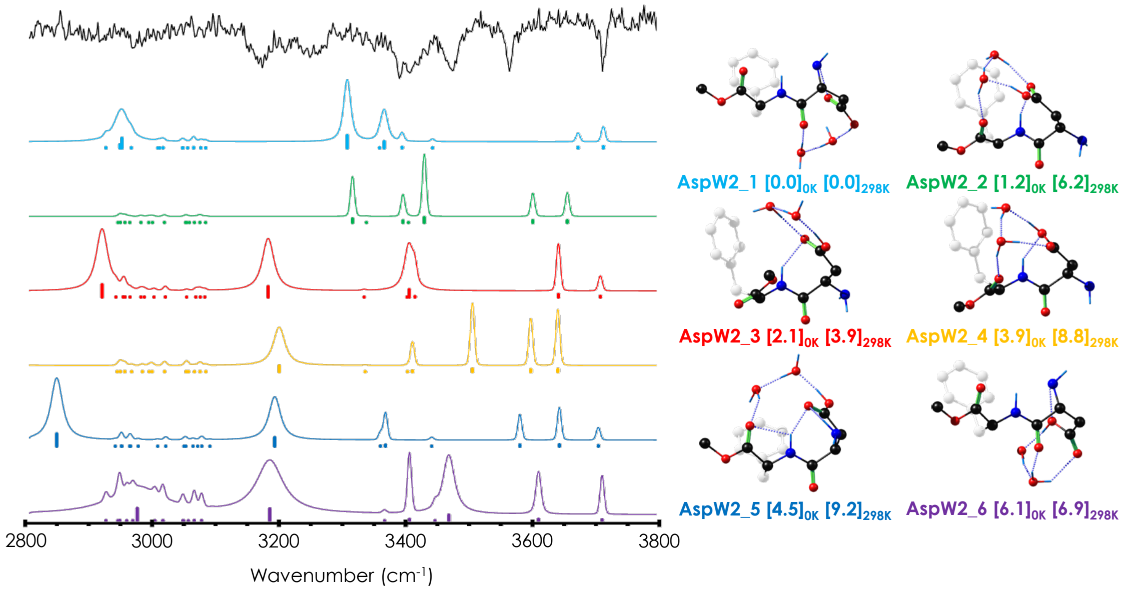

Figure S9. Experimental (black) and B3LYP/def2-VTZ (coloured) IR spectra for A_{W2} aggregate. Scaling factors 0.964, 0.9605, and 0.963 were used for OH, NH, and CH stretching vibrations, respectively, to account for anharmonicity.

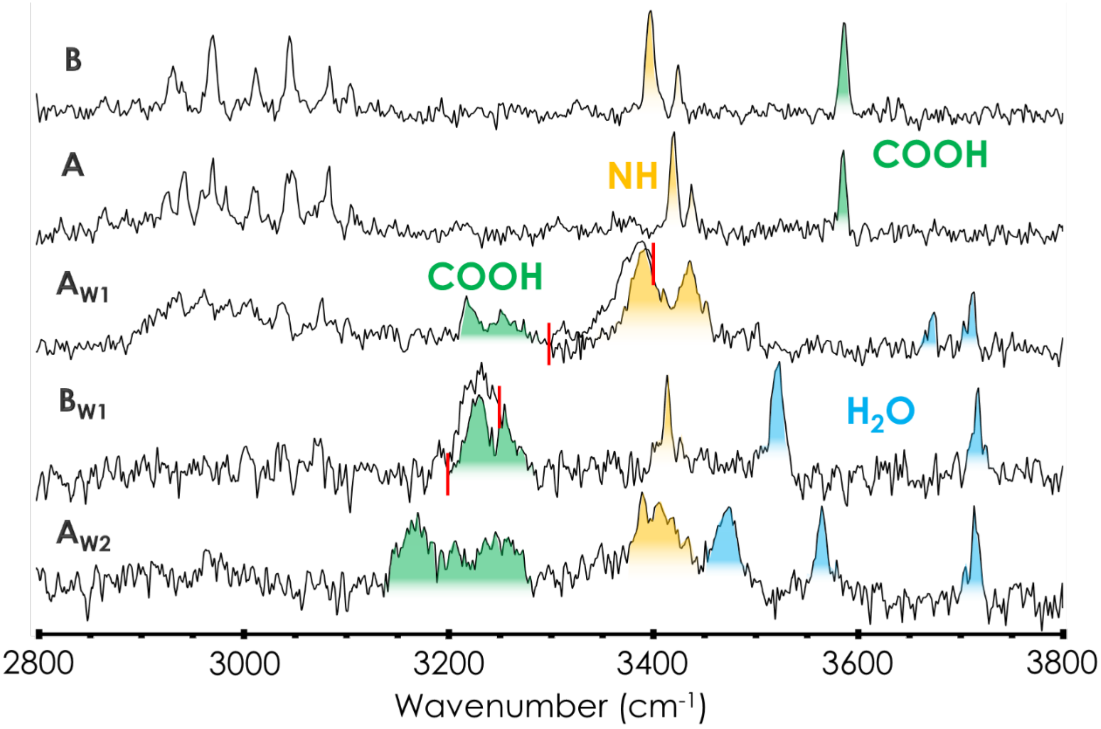

Figure S10. Summary of the experimental IRID spectra for aspartame·water$_{0\text{-}2}$ aggregates. The vertical red lines in the experimental traces mark the starting and ending point of different scans used to obtain the experimental data.

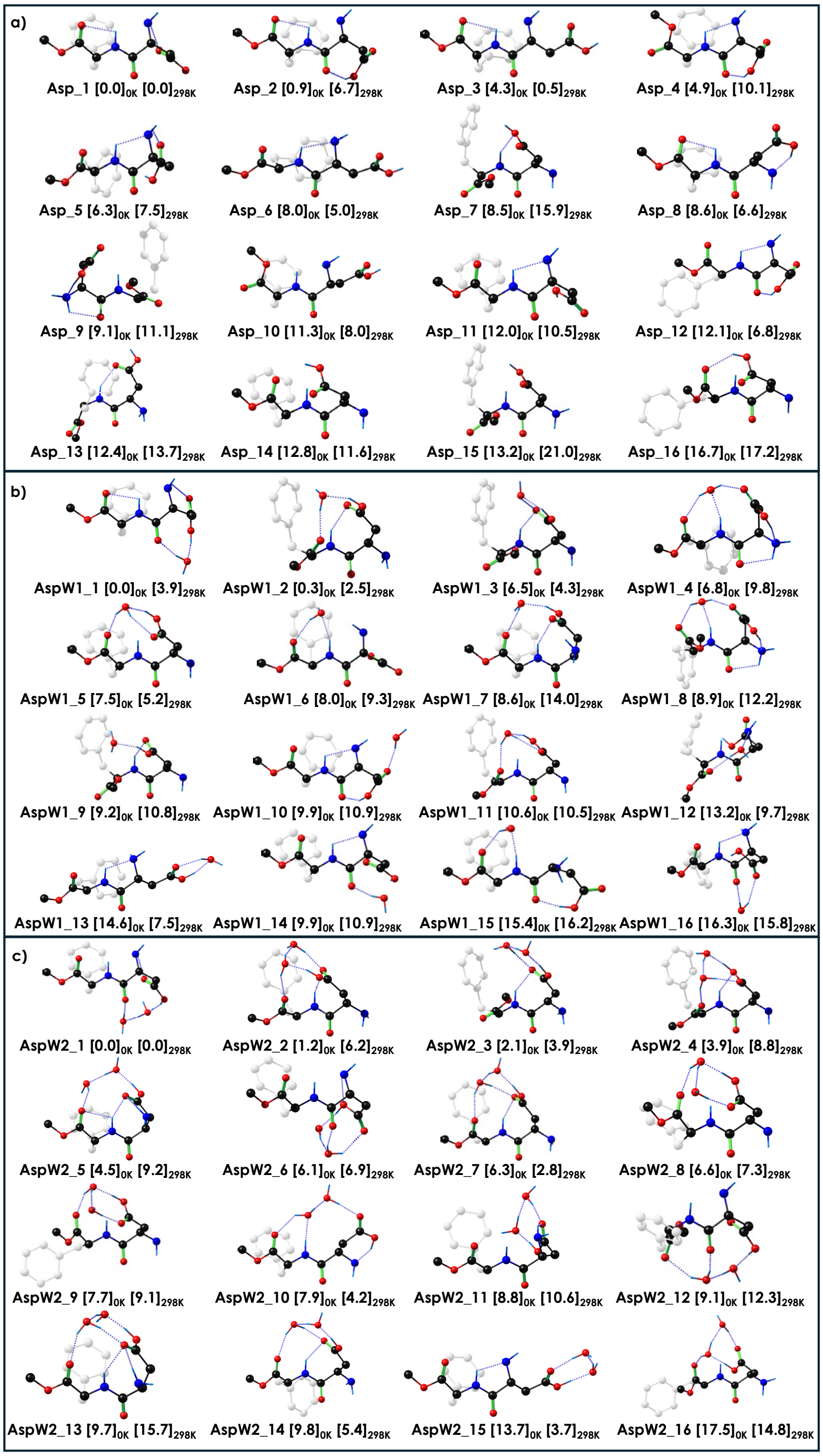

Figure S11: Some example structures resulting from the conformational search. For Asp (a), Asp_W1 (b), and Asp_W2 aggregates.
